# Regional Longitudinal Myocardial Deformation Provides Incremental Prognostic Information in Patients with ST-Segment Elevation Myocardial Infarction

**DOI:** 10.1371/journal.pone.0158280

**Published:** 2016-06-27

**Authors:** Tor Biering-Sørensen, Jan Skov Jensen, Sune H. Pedersen, Søren Galatius, Thomas Fritz-Hansen, Jan Bech, Flemming Javier Olsen, Rasmus Mogelvang

**Affiliations:** 1 Department of Cardiology, Herlev and Gentofte Hospital, University of Copenhagen, Copenhagen, Denmark; 2 Institute of Clinical Medicine, Faculty of Health Sciences, University of Copenhagen, Copenhagen, Denmark; University Hospital of Würzburg, GERMANY

## Abstract

**Background:**

Global longitudinal systolic strain (GLS) has recently been demonstrated to be a superior prognosticator to conventional echocardiographic measures in patients after myocardial infarction (MI). The aim of this study was to evaluate the prognostic value of regional longitudinal myocardial deformation in comparison to GLS, conventional echocardiography and clinical information.

**Method:**

In total 391 patients were admitted with ST-Segment elevation myocardial infarction (STEMI), treated with primary percutaneous coronary intervention and subsequently examined by echocardiography. All patients were examined by tissue Doppler imaging (TDI) and two-dimensional strain echocardiography (2DSE).

**Results:**

During a median-follow-up of 5.3 (IQR 2.5–6.1) years the primary endpoint (death, heart failure or a new MI) was reached by 145 (38.9%) patients. After adjustment for significant confounders (including conventional echocardiographic parameters) and culprit lesion, reduced longitudinal performance in the anterior septal and inferior myocardial regions (but not GLS) remained independent predictors of the combined outcome. Furthermore, inferior myocardial longitudinal deformation provided incremental prognostic information to clinical and conventional echocardiographic information (Harrell's c-statistics: 0.63 vs. 0.67, p = 0.032). In addition, impaired longitudinal deformation outside the culprit lesion perfusion region was significantly associated with an adverse outcome (p<0.05 for all deformation parameters).

**Conclusion:**

Regional longitudinal myocardial deformation measures, regardless if determined by TDI or 2DSE, are superior prognosticators to GLS. In addition, impaired longitudinal deformation in the inferior myocardial segment provides prognostic information over and above clinical and conventional echocardiographic risk factors. Furthermore, impaired longitudinal deformation outside the culprit lesion perfusion region seems to be a paramount marker of adverse outcome.

## Introduction

Early mechanical reperfusion and aggressive antiplatelet in combination with anticoagulant therapy have markedly improved outcome for patients with a ST-Segment elevation myocardial infarction (STEMI)[1]. However, in the aftermath patients are still in high risk of suffering yet another cardiovascular event. Consequently, efforts to clarify pathophysiological mechanisms, improve risk-stratification and identify targets for therapeutic intervention are very important.

Echocardiography after a myocardial infarction (MI) is a routine procedure for risk-stratification. Global left ventricle (LV) systolic function determined by LV end-systolic volume or LV ejection fraction (LVEF) has for several decades been the primary focus[2–4]. However, evaluating regional myocardial dysfunction by visually assessing the wall motion score index (WMSI), has been demonstrated to be superior to the volumetric measures of LV global function in regard to predict outcome following MI[5,6]. Novel echocardiographic techniques such as tissue Doppler imaging (TDI) and two-dimensional strain echocardiography (2DSE), which both provide objective measures of myocardial deformation, have in recent studies been demonstrated superior prognosticators to the conventional measures of global (LVEF) and regional (WMSI) LV systolic function[7–11]. In addition, the objective measures of evaluating myocardial deformation (TDI and 2DSE) are more reproducible and reliable than the WMSI which is based on visual estimation[12]. However, the studies which have evaluated the usefulness of the novel myocardial deformation measures in patient with MI have focused on the prognostic utility of only global longitudinal deformation parameters such as global strain and strain rate[7–11]. Nevertheless, almost a decade ago we were presented with the circumstance that evaluating regional myocardial dysfunction is superior to evaluating global function for risk-stratification strategies in MI patients[5,6]. So perhaps we can improve the usefulness of the novel myocardial deformation measures if evaluating regional in addition to global deformation performance.

The aim of this study was therefore to evaluate the prognostic value of the novel regional longitudinal myocardial deformation measures determined by TDI (systolic velocity and displacement) and 2DSE (strain and strain rate) and investigate if regional deformation parameters are superior to the global deformation parameter, Global Longitudinal Strain (GLS), in predicting outcome in patients with STEMI treated with primary percutaneous coronary intervention (pPCI).

## Methods

### Study population

From September 2006 to December 2008 a total of 391 patients were admitted with a STEMI, treated with pPCI, and underwent a detailed echocardiographic examination at Gentofte Hospital, University of Copenhagen, Denmark. All patients were prospectively included in the present study. Four patients were excluded due to inadequate quality of the echocardiographic examination and 14 due to atrial fibrillation. The study population has previously been described[13,14].

### Echocardiography

Echocardiography was performed using Vivid 7 ultrasound systems (GE Healthcare, Horten Norway) with a 3.5-MHz transducer by experienced sonographers. The echocardiographic examinations were performed after the STEMI (median 2 (IQR: 1–3) days). All participants were examined with conventional two-dimensional echocardiography, pulsed-wave and color TDI. All echocardiograms were stored digitally and analysed off-line using commercially available software (EchoPac, GE Healthcare, Horten Norway) by a single investigator, who was blinded to all other patient data.

#### Conventional echocardiography

LV end-diastolic dimensions (interventricular septum wall thickness, LV internal dimension, and LV posterior wall thickness) were obtained from the parasternal long-axis view at the mitral valve leaflet tips and used to calculate the LV mass index (LVMI)[15]. Pulsed wave Doppler at the apical position was used to record mitral inflow between the tips of the mitral leaflets. Peak velocity of early (E) and atrial (A) diastolic filling and deceleration time of the E-wave (DT) were measured and the E/A-ratio was calculated. LV end-diastolic volume, end-systolic volume and the LVEF were determined using modified biplane Simpson’s method. Wall motion index score was assessed using the 16 segment model[15]. Left atrial volume was estimated by the area-length method and divided with BSA creating the Left Atrial Volume Index (LAVI)[15].

#### Tissue Doppler Imaging

Pulsed-wave TDI tracings were obtained with the range gate placed at the septal and lateral mitral annular segments in the 4-chamber view. The peak longitudinal early diastolic (e’) velocity was measured and the average was calculated from the lateral and septal velocities and used to obtain the E/e’. Diastolic function was assessed using mitral inflow velocity profiles and pulsed-wave TDI tracings from the septal and lateral mitral annulus according to the present guidelines[16].

Color TDI loops were obtained in the apical 4-chamber, 2-chamber and apical long-axis view at the highest possible frame rate (mean 169 frames/s, standard deviation (SD) 33 frames/s). Peak longitudinal systolic (s’) velocities were measured at the 6 mitral annular (MA) sites dividing the LV into 6 regions of interest: The septal, lateral, anterior, inferior, posterior and anterior septal myocardial wall. Integration of the systolic color TDI velocity curves from the 6 regions of interest allowed for the assessment of systolic MA longitudinal displacement (LD)[17]. The systolic MA LD was measured within the ejection phase (between aorta valve opening (AVO) and closing times (AVC)[13,18]), thereby excluding any postsystolic shortening from the systolic MA LD measurements.

#### Two-dimensional strain echocardiography

2DSE was performed from the apical 4-chamber, 2-chamber and apical long-axis view (mean 86 frames/s, SD 23 frames/s). By speckle tracking, the endocardial border was traced in end systole. The integrity of speckle tracking was automatically detected and visually ascertained. In case of poor tracking, the region of interest tracing was readjusted. Segments with persistent inadequate tracking were excluded from analysis (14% of all segments). Segmental longitudinal peak systolic strain was measured in all views between aortic valve opening and closing for the 6 basal, 6 midventricular and 6 apical segments, and averaged to provide GLS. Furthermore, in each segment peak longitudinal systolic strain rate (SRs)(a measure of deformation velocity) were measured. The regional strain and SRs were calculated for the septal, lateral, anterior, inferior, posterior and anterior septal myocardial walls by averaging the basal, midventricular and apical measures in each myocardial wall. We, in accordance with other groups, have previously demonstrated good reproducibility of all deformation measures (both obtained by TDI[17,19] and speckle tracking[20,21]) in patients with ischemic heart disease.

### Primary PCI procedure

Glycoprotein IIb/IIIa inhibitors were used at the discretion of the operator. Culprit lesion was designated by the operator. Multivessel disease was defined as 2- or 3 vessel disease and complex lesions as type C-lesions. Subsequent medical treatment included anti-ischemic, lipid-lowering and anti-thrombotic drugs according to current treatment guidelines.

### Follow-up and end points

The primary endpoint was the combined endpoint of all-cause mortality, a new MI (re-MI) or admission due to Heart Failure (CHF). Follow-up was 100%. Follow-up data on re-MI and admission with CHF were obtained from the Danish National Board of Health’s National Patient Registry, using ICD-10 codes. Follow-up data on mortality were collected from the National Person Identification Registry.

### Statistics

Proportions were compared using χ^2^-test, continuous Gaussian distributed variables with Student’s t-test and Mann-Whitney test if non-Gaussian distributed.

Hazards ratios (HR) were calculated by Cox proportional hazards regression analyses. The assumptions of linearity and proportional hazards in the models were tested. Harrell’s c-statistics[22] were calculated in order to test the prognostic performance of the different measures of longitudinal performance from the different myocardial segments. Analysis of variance (ANOVA) was performed to test if the regional TDI and 2DSE values varied according to the culprit lesion. If the ANOVA was found to be significant Bonferroni correction was performed in the comparison between individual segments. A p-value ≤ 0.05 in 2-sided test was considered statistically significant. All analyses were performed with STATA Statistics/Data analysis, SE 12.0 (StataCorp, Texas,USA).

All participants provided written informed consent to participate in this study and all aspects of the study was approved by the local scientific ethical committee for the region of Copenhagen (Region Hovedstaden) and The Danish Data Protection Agency, and complied with the 2^nd^ Declaration of Helsinki and later amendments.

## Results

Baseline characteristics are displayed in *Table 1*.

**Table 1:** Baseline clinical characteristics in ST-segment elevation myocardial infarction (STEMI) patients treated by primary Percutaneous Coronary Intervention (pPCI) stratified according to major adverse outcome.

	No major adverse outcome (n = 228)	Major adverse outcome (n = 145)	P-value
Age (years)	60±11	65±12	*<0*.*001*
Male gender	75%	74%	*0*.*84*
MAP (mmHg)	99±17	101±20	*0*.*29*
Hypertension	32%	32%	*0*.*87*
Diabetes	6%	13%	*0*.*013*
Current smoker	50%	54%	*0*.*53*
Hypercholesterolemia	17%	17%	*0*.*98*
Previous AMI	4%	6%	*0*.*22*
BMI (kg/m^2^)	27.1±4.2	26.2±4.7	*0*.*05*
Peak Troponin I (μg/L)	91 (25–207)	135 (36–287)	*0*.*016*
eGFR^c^	75.7±20.3	71.8±23.4	*0*.*09*
Symptom-to-balloon time (min)	185 (120–303)	190 (135–315)	*0*.*54*
Complex lesion	41%	52%	*0*.*047*
Multivesseldisease	25%	31%	*0*.*24*
LAD Culprit lesion	47%	48%	*0*.*86*
RCA Culprit lesion	39%	43%	*0*.*81*
LCx Culprit lesion	14%	8%	*0*.*07*
Glycoprotein IIb/IIIainhibitor	22%	27%	*0*.*32*
TIMI grade flow before pPCI			
TIMI 0 flow	61%	65%	
TIMI 1 flow	13%	14%	*0*.*29*
TIMI 2 flow	10%	12%	
TIMI 3 flow	17%	10%	
TIMI grade flow after pPCI			
TIMI 0 flow	4%	3%	
TIMI 1 flow	2%	8%	*0*.*05*
TIMI 2 flow	8%	8%	
TIMI 3 flow	85%	81%	
LVEF (%)	48±8	43±9	*<0*.*001*
WMSI	1.5 (1.3–1.8)	1.7 (1.4–2.1)	*<0*.*001*
LVEDV/BSA (mL/m^2^)	49 (41–58)	49 (41–59)	*0*.*81*
LVESV/BSA (mL/m^2^)	25 (21–31)	27 (22–36)	*0*.*09*
LVMI (g/m^2^)	89.1 (73.0–106.8)	93.2 (79.2–112.2)	*0*.*034*
LAVI (ml/m^2^)	25±7	24±7	*0*.*34*
E (m/s)	0.77±0.18	0.76±0.21	*0*.*82*
A (m/s)	0.74±0.19	0.76±0.21	*0*.*30*
E/A ratio	1.05 (0.85–1.30)	0.98 (0.82–1.31)	*0*.*25*
DT (ms)	200±53	198±59	*0*.*73*
E/e’	10.3 (8.1–12.1)	10.9 (8.9–14.2)	*0*.*003*
Grade of diastolic function			
Normal	34%	19%	
Grade I dysfunction	21%	26%	*0*.*001*
Grade II dysfunction	42%	43%	
Grade III dysfunction	4%	12%	
GLS (%)	12.7±4.1	10.7±3.8	*<0*.*001*

Continuous Gaussian distributed variables are represented by the mean and standard deviation. Continuous non-Gaussian distributed variables are represented by the median and interquartile range. Categorical variables are represented by proportions. MAP = Mean Arterial Blood Pressure, AMI = Acute Myocardial Infarction, BMI = Body Mass Index, eGFR = estimated glomerular filtration rate, LAD = Left Anterior Descending coronary artery, RCA = Right coronary artery, LCx = Left Circumflex coronary artery, LVEF = Left Ventricular Ejection Fraction, WMSI = Wall Motion Score Index, LVId = Left Ventricular Internal Diameter in Diastole, LVEDV = Left Ventricular End-Diastolic Volume, LVEDV = Left Ventricular End-Systolic Volume, LVMI = Left Ventricular Mass Index, Left Atrial Volume Index = LAVI, E = peak transmitral early diastolic inflow velocity, A = peak transmitral late diastolic inflow velocity, DT = deceleration time of early diastolic inflow, TIMI = Thrombolysis in Myocardial Infarction classification, e’ = average peak early diastolic longitudinal mitral annular velocity determined by pulsed-wave TDI, GLS = Global longitudinal peak systolic strain.

In the course of follow-up (median 64, IQR: 30–73 months), 51 (13.7%) patients were admitted due to re-MI, 70 (18.8%) patients were admitted to hospital due to CHF and 59 (15.8%) patients died. The combined endpoint was reached by 145 (38.9%) patients.

### Regional longitudinal deformation and prognosis

**Fig 1:**
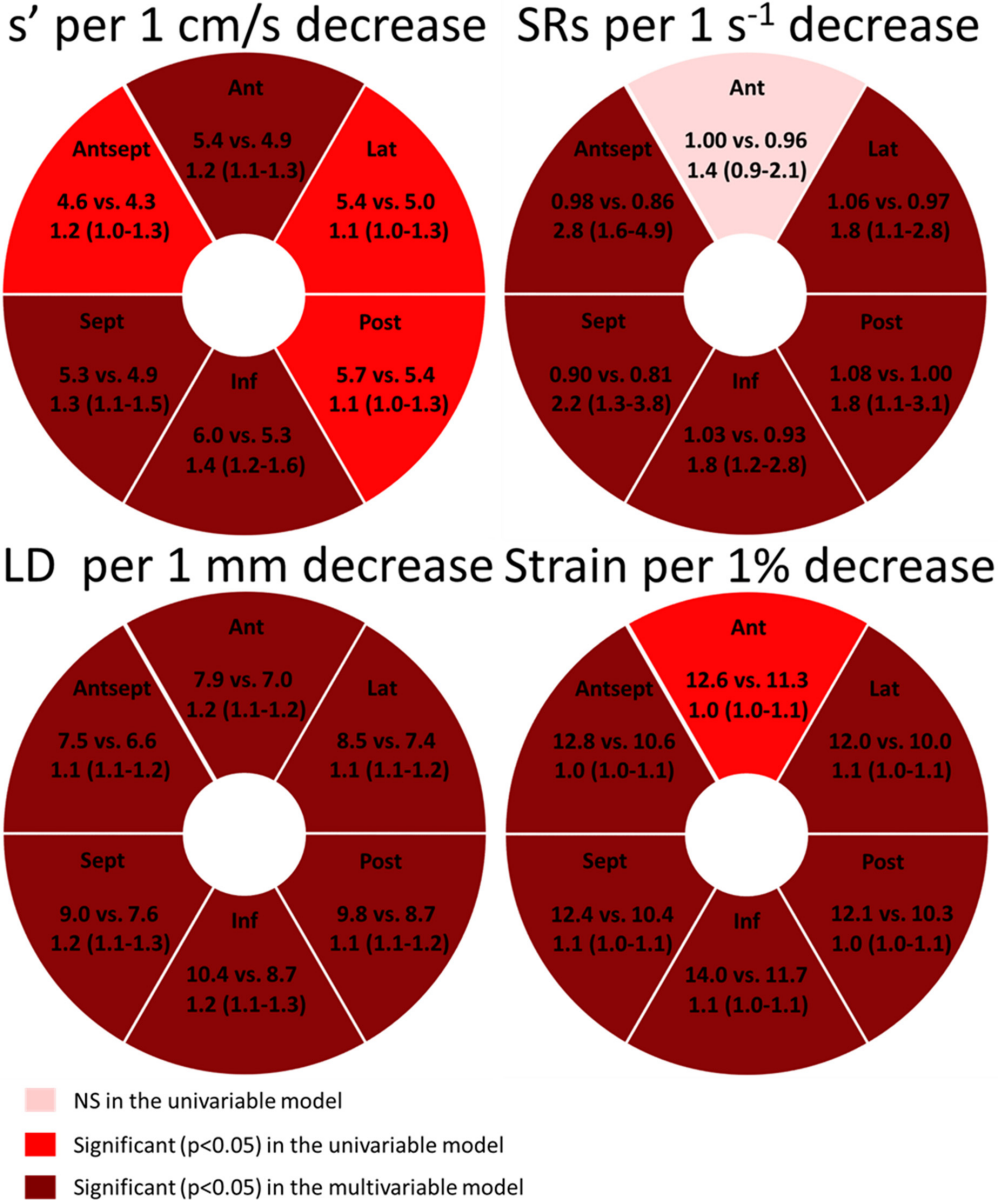
Regional longitudinal deformation and prognosis. Univariable and multivariable Cox proportional hazards regression models describing the risk of an adverse outcome per 1 decrease in longitudinal performance in each segment described by the TDI parameters and the 2DSE parameters, respectively. Depicting the mean value of the longitudinal deformation parameters in patients without an adverse outcome vs. patients with an adverse outcome, and the hazard ratio (95% confidence intervals) of adverse outcome associated with 1 decrease in longitudinal performance. In the multivariable models, the Regional longitudinal deformation measures are adjusted for age, gender, diabetes, complex lesion, peak TnI and culprit lesion. TDI = Tissue Doppler Imaging, 2DSE = Two-dimensional strain echocardiography, LD = Mitral annular longitudinal displacement determined by color TDI, s’ = Peak systolic longitudinal mitral annular velocity determined by color TDI, SRs = Peak longitudinal systolic strain rate, LVEF = Left Ventricular Ejection Fraction, LVMI = Left Ventricular Mass Index. ANT = Anterior, LAT = Lateral, POST = Posterior, INF = Inferior, SEPT = Septal, ANT SEPT = Anterior septal.

The numerical values of all the regional deformation parameters obtained by TDI (s’ and LD) and 2DSE (strain and SRs) were tested as predictors of an adverse outcome (*Fig 1*). In univariable and multivariable Cox proportional hazards regression models, a decrease in longitudinal performance in almost all regions determined by all deformation parameters, regardless if obtained by TDI or 2DSE, were significantly associated with an increased risk of an adverse outcome (*Fig 1*). However, after adjusting for age, gender, peak TnI, diabetes, complex lesion, LVEF, diastolic dysfunction, LVMI and culprit lesion, a pattern appeared. Decreasing longitudinal performance in the anterior septal and inferior myocardial regions seemed to be superior prognostic markers, since longitudinal deformation parameters (LD assessed by TDI, together with strain and SRs assessed by 2DSE) obtained from these myocardial walls remained as independent predictors of the combined outcome (Inferior LD: HR 1.12 (1.03–1.22), p = 0.011, per 1 mm decrease; inferior strain: HR 1.04 (1.00–1.07), p = 0.043, per 1% decrease; anterior septal SRs: HR 2.07 (1.05–4.09), p = 0.036, per 1 s^-1^ decrease). In comparison, GLS did not remain a significant independent predictor after adjustment for the same confounders (HR: 1.05 (1.00–1.12), p = 0.06; per 1% decrease).

**Fig 2:**
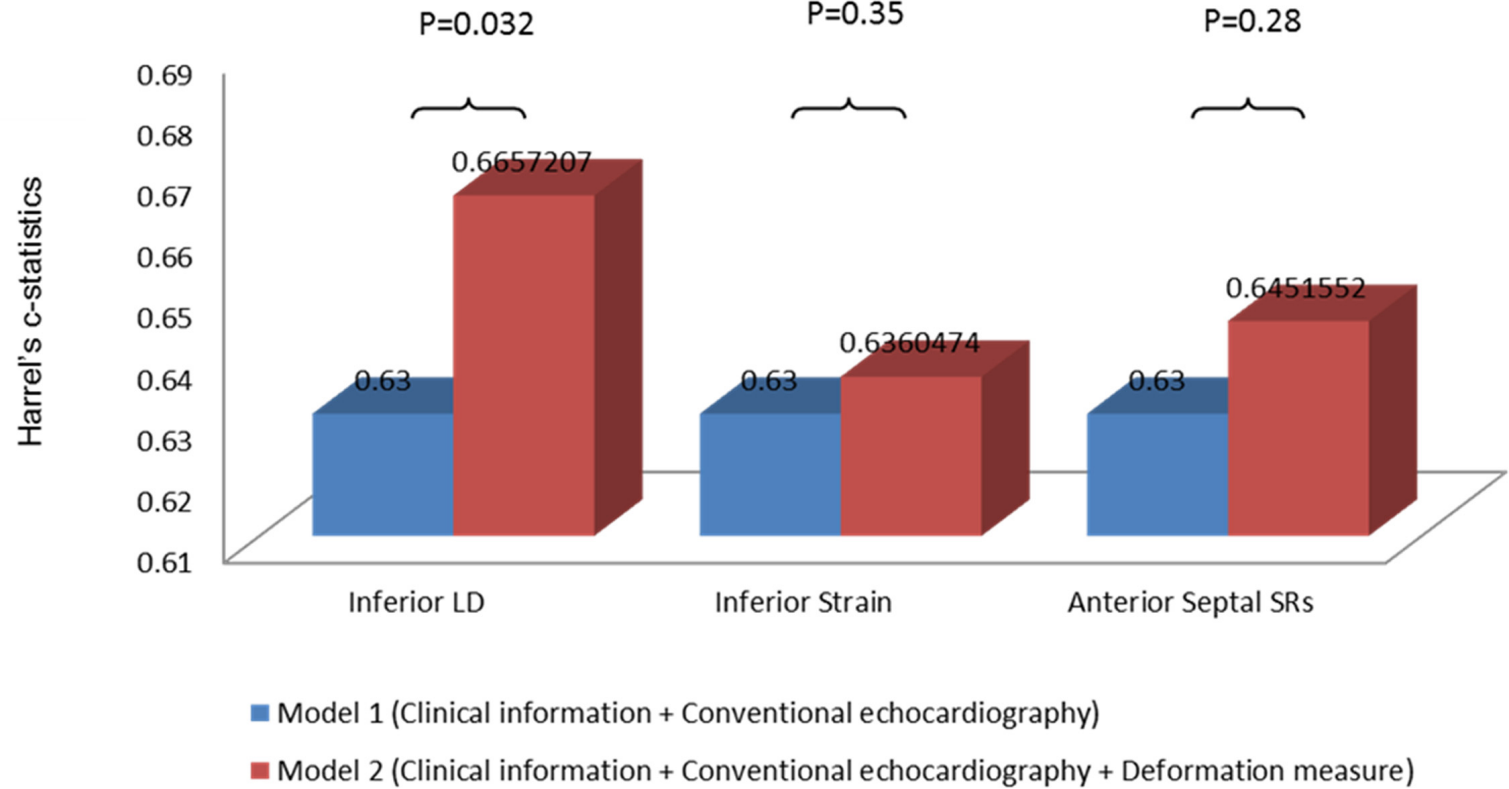
Incremental prognostic information by adding an assessment of the Regional longitudinal deformation. The Harrell's c-statistic values obtained from the multivariable Cox proportional hazards regression models. Model 1 includes age, gender, peak TnI, systolic dysfunction determined by an LVEF<45 and/or abnormal diastolic function determined by the presence of diastolic dysfunction grade 1 to 3. Model 2 includes model 1 and one of the longitudinal deformation measures described by the TDI parameters or the 2DSE parameters. TDI = Tissue Doppler Imaging, 2DSE = Two-dimensional strain echocardiography, LD = Mitral annular longitudinal displacement determined by color TDI, SRs = Peak longitudinal systolic strain rate.

When testing if the longitudinal deformation parameters which provide significant independent prognostic information (LD assessed by TDI, together with strain and SRs assessed by 2DSE) also provide prognostic information beyond clinical and conventional echocardiographic predictors, only LD obtained from the inferior myocardial wall provided incremental prognostic information (*Fig 2*), determined by a significant increase in the Harrell's c-statistics.

### Regional longitudinal deformation and culprit lesion

**Fig 3:**
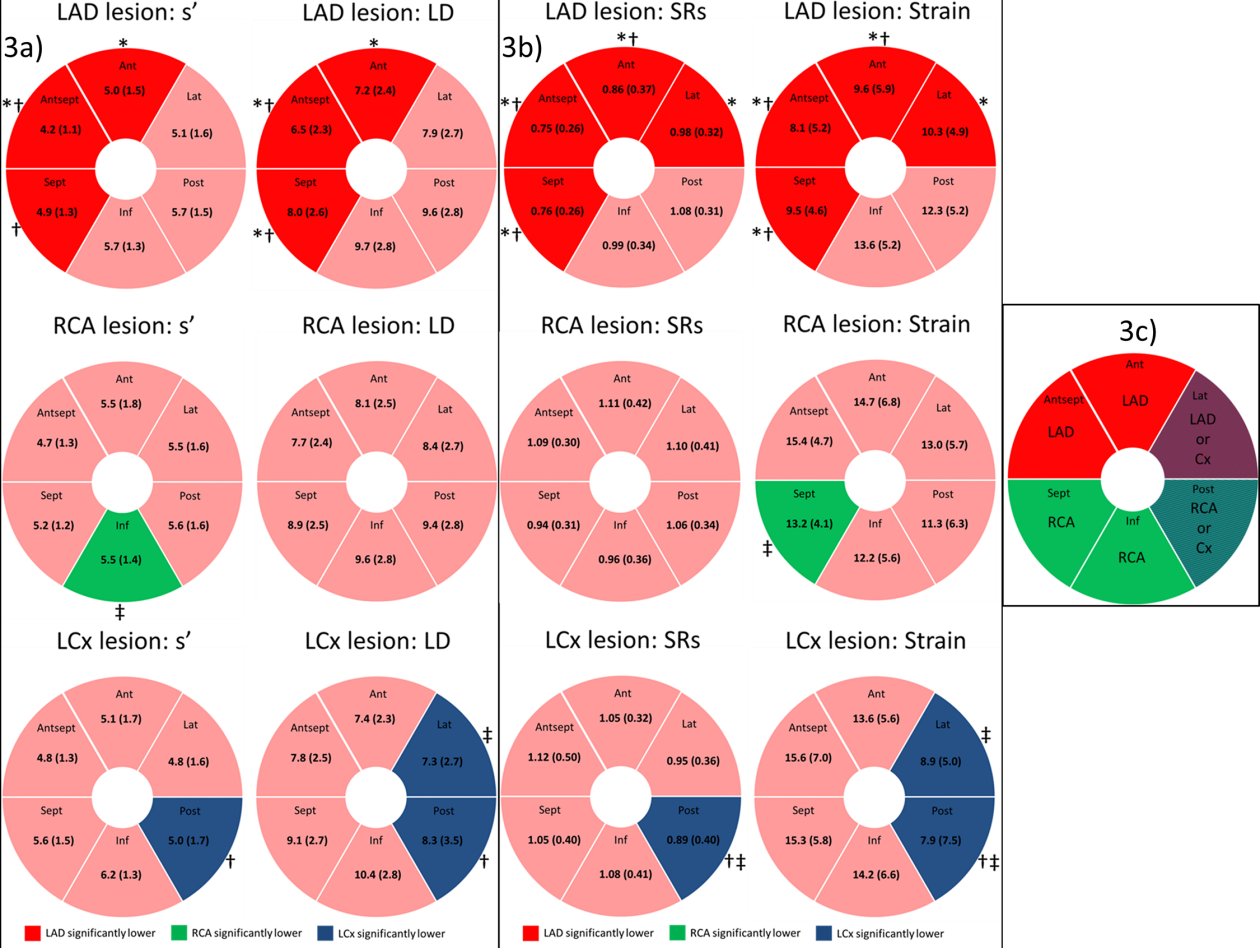
Regional longitudinal deformation and culprit lesion. Patients stratified according to the location of their culprit lesion in the left anterior descending (LAD), the right coronary artery (RCA) or the left circumflex coronary artery (LCx). *Fig 3A*: The mean value of the TDI parameters at all six myocardial walls for patients stratified according to the location of their culprit lesion. *Fig 3B*: The mean value of the 2DSE parameters at all six myocardial walls for patients stratified according to the location of their culprit lesion. *Fig 3C*: The typical distribution of coronary artery blood supply to the 6 myocardial walls is displayed[15]. * indicates a p-value < 0.05 (Bonferroni corrected) when comparing LAD lesions with RCA lesions. † indicates a p-value < 0.05 (Bonferroni corrected) when comparing LAD lesions with Cx lesions. ‡ indicates a p-value < 0.05 (Bonferroni corrected) when comparing RCA lesions with Cx lesions. Values represent mean (±SD). LAD = Left Anterior Descending coronary artery, RCA = Right coronary artery, LCx = Left Circumflex coronary artery, s’ = peak systolic longitudinal mitral annular velocity determined by color Tissue Doppler Imaging, LD = Mitral annular longitudinal displacement determined by color TDI, SRs = Peak longitudinal systolic strain rate, ANT = Anterior, LAT = Lateral, POST = Posterior, INF = Inferior, SEPT = Septal, ANT SEPT = Anterior septal.

Patients were stratified according to the location of their culprit lesion in the left anterior descending (LAD), the right coronary artery (RCA) or the left circumflex coronary artery (LCx). The mean s’ and LD (obtained by TDI), and mean SRs and strain (obtained by 2DSE) value at all six myocardial walls was calculated (Fig 3). Patients with LAD lesions had significantly lower s’ and LD in the anterior septal myocardial wall compared to patients with both RCA and LCx lesions. Additionally, s’ and LD were lower in the anterior myocardial wall in patients with LAD lesions compared to patients with RCA lesions and lower s’ in the septal myocardial wall compared to patients with LCx lesions. Furthermore, patients with LAD lesions had significantly lower LD values in the septal myocardial wall compared to patients with both RCA and LCx lesions. Patients with RCA lesions had significant lower s’ in the inferior myocardial wall compared to patients with LCx lesions and patients with an LCx lesions had lower s’ and LD values in the posterior myocardial wall compared to patients with LAD lesions. In addition, patients with LCx lesions had significantly lower LD values in the lateral myocardial wall compared to patients with RCA lesions.

Patients with LAD lesions had significantly lower SRs and strain in the septal, anteroseptal and anterior myocardial walls compared to patients with RCA and LCx lesions. Furthermore, they had significantly lower SRs and strain in the lateral myocardial wall compared to patients with RCA lesions. In addition, patients with RCA lesions had significantly lower strain values in the septal myocardial wall compared to patients with LCx lesions. Patients with LCx lesions had significantly lower SRs and strain in the posterior myocardial wall compared to patients with RCA and LAD lesions. Additionally, patients with LCx lesions had significantly lower strain values in the lateral myocardial wall compared to patients with RCA lesions.

### Regional longitudinal deformation patterns, culprit lesion and prognosis

**Fig 4:**
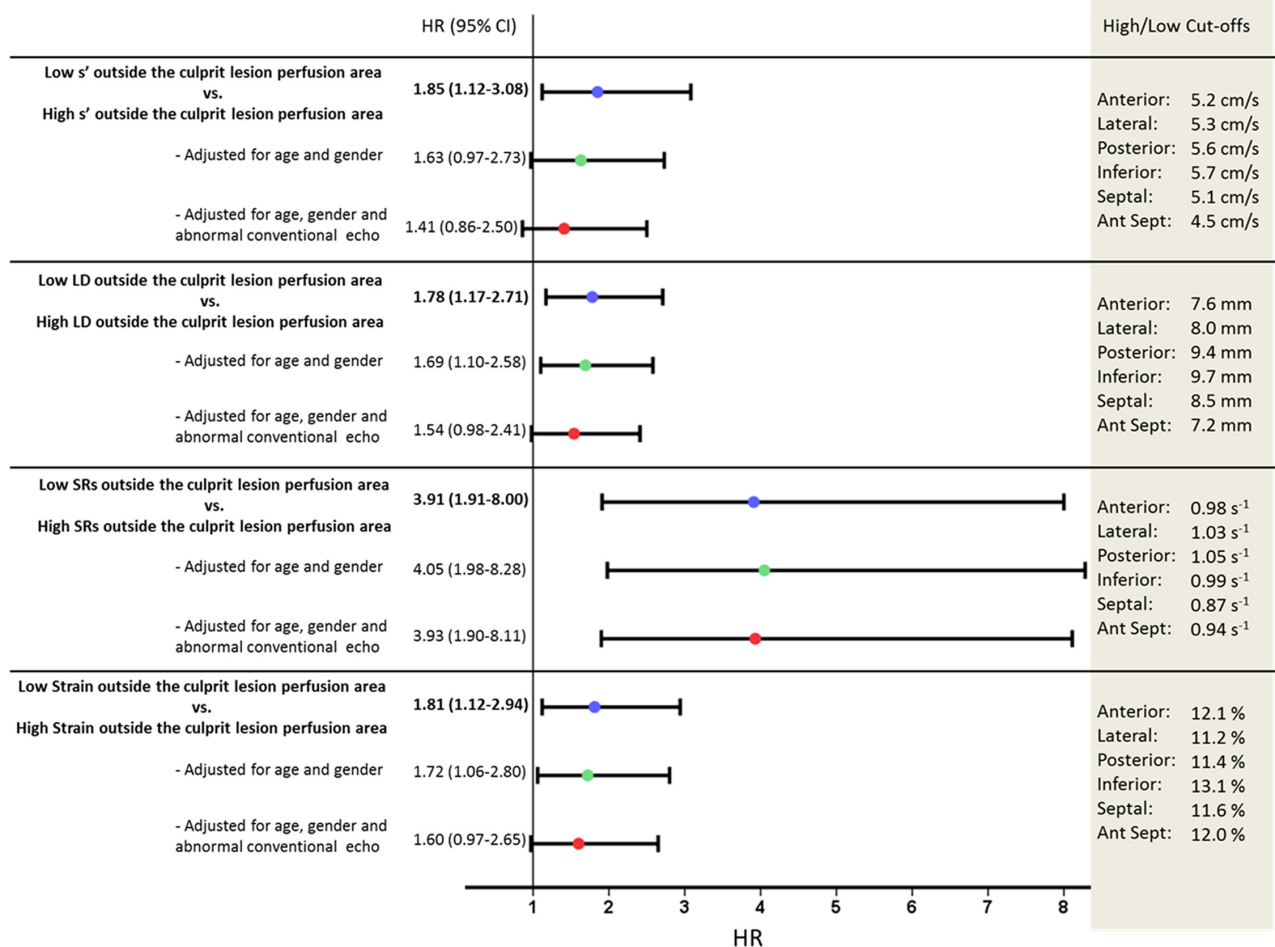
Regional longitudinal deformation outside the culprit perfusion region and prognosis. Univariable and multivariable Cox proportional hazards regression models describing the risk of an adverse outcome for patients stratified into high or low regional longitudinal deformation parameters outside the culprit perfusion region. An abnormal conventional echocardiography is defined by systolic dysfunction determined by an LVEF<45 and/or abnormal diastolic function determined by the presence of diastolic dysfunction grade 1 to 3. Depicting the hazard ratio and the 95% confidence intervals. Also depicting the cut-off values, which are obtained from the mean values, for each regional longitudinal deformation parameter in each myocardial wall. s’ = Peak systolic longitudinal mitral annular velocity determined by color Tissue Doppler Imaging, LD = Mitral annular longitudinal displacement determined by color Tissue Doppler Imaging, SRs = Peak longitudinal systolic strain rate.

We assigned the segments to each vascular territory in order to comply optimally with the guidelines[15]: The left anterior descending coronary artery territory included the anterior and the anterior septal myocardial regions; the left circumflex coronary artery territory included the lateral and posterior myocardial regions; and the right coronary artery territory included the inferior and septal myocardial regions. Patients were hereafter stratified into two groups defined by having high or low regional LD, s’, strain or SRs outside the culprit lesion perfusion area, respectively. The cut-off values for high or low regional longitudinal deformation were obtained from the mean values, for each regional longitudinal deformation parameter in each myocardial wall. Patients with impaired longitudinal deformation outside the culprit perfusion region determined by TDI, defined by one or more segments with low s’ and LD, had approximately two times the risk of being admitted a new MI, CHF or dying, than patients without low s’ and LD outside the culprit lesion perfusion area, respectively (*Fig 4*). Patients with impaired longitudinal deformation outside the culprit perfusion region determined by 2DSE, defined by one or more segments with low SRs or strain, had approximately four or two times the risk of being admitted with an adverse event, compared to patients without low SRs or strain outside the culprit lesion perfusion area, respectively (*Fig 4*). After multivariable adjustment for age, gender, systolic dysfunction (determined by an LVEF<45) and/or abnormal diastolic function (determined by the presence of diastolic dysfunction grade 1 to 3), only a pattern of low SRs outside the culprit lesion perfusion area remained a significant prognosticator.

## Discussion

The present prospective study of STEMI patients is the first to demonstrate, that the regional longitudinal myocardial deformation measures (regardless if determined by TDI or 2DSE), seem superior prognosticators compared to global longitudinal myocardial deformation measure, GLS.

Decreasing longitudinal performance in the anterior septal and inferior myocardial walls appeared to be superior prognostic markers, since the measured longitudinal deformation parameters obtained from these regions remained as independent predictors of the combined outcome. Furthermore, impaired longitudinal performance in the inferior myocardial region, determined by LD, provides prognostic information over and above clinical and conventional echocardiographic risk factors (*Fig 2*). In addition, an impaired longitudinal deformation performance outside the culprit lesion perfusion region seems to be a paramount marker of an adverse outcome (*Fig 4*).

### Regional longitudinal deformation and prognosis

The myocardial fibres most susceptible to ischemia are the longitudinally orientated fibers which are located subendocardially[23]. Measurements of longitudinal motion and deformation are therefore the most sensitive markers of ailing myocardium in the setting of acute MI. Myocardial ischemia and infarction decreases regional wall motion and deformation[24], regardless if measuring myocardial tissue velocities, displacement, strain or strain rate[20,25]. This causes a decrease in peak systolic deformation and an increase in the post systolic deformation causing an ineffective contraction in the affected segment[24]. Until now, only the prognostic significance of global longitudinal performance (GLS) has been investigated as a parameter of longitudinal deformation performance after an MI[7,8,10]. However, the results from the present study indicate that we may overlook or dilute the prognostic performance of using longitudinal deformation as prognostic markers following a STEMI, if we only evaluate the global longitudinal deformation, since impaired longitudinal deformation in specific myocardial walls seems more severe than in others. Likewise, a recent study demonstrated that regional deformation as assessed by 3D speckle tracking echocardiography may be more accurate in estimating LV myocardial scaring and the extent of transmural infarction following a STEMI[26]. We found that decreasing longitudinal deformation in the anterior septal and inferior myocardial walls were paramount markers of an adverse prognosis, irrespective of culprit lesion and regardless if measured by 2DSE or TDI (*Figs 1, 2*and *4*). These findings may be explained by the circumstance that the longitudinal muscle fibers stretching from the anterior septal to the inferior myocardial walls are the myocytes in the left ventricle which counterbalances the highest wall stress in the left ventricle, due to the greater radius of curvature of the myocardial walls[27]. Therefore, when these myocytes which preform the highest workload of all longitudinally oriented myocardial fibres in the left ventricle are attenuated following a STEMI the prognosis is adverse. Additionally, it has been hypothesized that the inferio-posterior region of the LV is innervated by greater parasympathetic (vagal) afferents, and an ailing myocardium in this region may alter or destroy the parasympathetic fibers, leading to autonomic dysfunction of the heart with higher arrhythmic potential [28]. Interestingly, in accordance with this, we have recently demonstrated that impaired longitudinal deformation in the inferior myocardial wall as assessed by TDI was the strongest predictor of ventricular arrhythmias and cardiovascular mortality in patients with ischemic cardiomyopathy[29]. Furthermore, in patients suffering from stroke, which is well known to cause an increased sympathetic tone[30] and impaired parasympathetic function[31] both of which are predisposing factors for sudden cardiac death[32], impaired longitudinal function of the inferior wall has been demonstrated to be the strongest echocardiographic predictor of mortality[33]. Therefore, impaired myocardial function in different regions of the LV might confer different risk profiles following STEMI. However, whether these differences are caused by differences in pro-arrhythmic potential or resting state workload of the myocardial fibers is not known. In contrast, when averaging the longitudinal performance of all the myocardial walls in one parameter, GLS, an evaluation of longitudinal performance did not provide independent or incremental information about the risk of an adverse outcome, when the conventional risk factors were taken into account. The previous studies evaluating the GLS included patients with both non-ST-segment elevation MI and STEMI patients[7,10], treated with either thrombolytic therapy or PCI[10]. This makes our population more homogeneous and less confounded by difference in therapy. Furthermore, Ersbøl and colleagues found GLS to be a superior prognostic marker in low risk MI patients (only 6.7% reached the combined endpoint) with a near normal LVEF (above 40%)[7]. These differences in study design might explain the discrepancy in the predictive power of GLS between our and previous studies[7,10]. The only previous study evaluating GLS in high risk STEMI patients, found that GLS was not superior to conventional echocardiography in predicting outcome[8]. However, no previous studies have compared prognostic significance of regional longitudinal deformation to global longitudinal deformation.

When comparing the different methods of obtaining information about longitudinal deformation, the single strongest parameter seems to be the LD, since the MA LD obtained from the inferior myocardial wall remained a statistically significant independent predictor and provided incremental prognostic information over and above clinical and conventional echocardiographic risk factors (*Fig 2*). MA LD obtained by TDI has recently been demonstrated to be a very accurate measure of the mitral annular plane systolic excursion (MAPSE) obtained by M-mode echocardiography[17]. Thus, perhaps the conventional MAPSE, the oldest method of evaluating LV longitudinal function, is just as (or more) appropriate as the novel deformation measures for risk stratification in patients following an MI.

### Regional longitudinal deformation patterns, culprit lesion and prognosis

Another advantage of performing an evaluation of the regional longitudinal deformation performance in STEMI patients is the possibility to evaluate the longitudinal deformation within and outside the culprit lesion perfusion region (*Figs 3*and *4*).

The regional 2DSE parameters seemed more sensitive markers of acute ischemia than the regional TDI parameters, since the 2DSE parameters were more closely associated with the culprit lesion perfusion region (*Fig 3*). This may be due to the fact that regional myocardial velocities obtained by TDI have the disadvantage of being influenced both by tethering to adjacent segments and overall heart movement[34], which makes 2DSE more suitable for diagnosing impairment of the regional longitudinal mechanics caused by ischemia[20].

Patients with impaired longitudinal deformation outside the culprit perfusion region determined by TDI or 2DSE are in significantly higher risk of being admitted due to re-MI or CHF, or dying, than patients without impaired longitudinal deformation outside the culprit lesion perfusion area (*Fig 4*).

Low SRs outside the culprit perfusion region seem to be the gravest marker of an adverse outcome (*Fig 4*). This is probably due to the fact that SRs has been demonstrated to be the most accurate marker of regional myocardial contractile function[35]. Therefore, patients with low SRs outside the culprit perfusion region lack the ability of a compensatory increase in contractile function in the unaffected regions.

### Limitations

We did not investigate the cause of death. However, we assume that individuals with cardiac dysfunction determined by echocardiography following a STEMI are more likely to die due to cardiovascular causes and that, if we were able to limit our analysis to cardiovascular deaths, the prognostic impact of impaired deformation parameters, regardless if determined by TDI or 2DSE, would be even greater.

Additionally, 2DSE was not performed in the parasternal short axis view why we did not obtain transmural or radial strain. Nevertheless, previous studies assessing the prognostic utility of myocardial deformation in MI patients have primarily focused on longitudinal deformation[7,9,14] since the longitudinal fibers, primarily located in the sub-endocardial region, are most vulnerable to ischemia[23].

Unfortunately, we did not assess coronary dominance in the present report which is a limitation. However, it is known that approximately 70% of the general population is right-dominant, 20% is co-dominant, and 10% is left-dominant, why the vast majority is expected to be right-dominant.

Furthermore, the risk of residual confounders always exists in a non-randomized study.

## Conclusion

The regional longitudinal myocardial deformation measures, regardless if determined by TDI or 2DSE, seem superior prognosticators than the global longitudinal myocardial deformation measure, GLS. In addition, impaired longitudinal deformation in the inferior myocardial wall appeared to be a superior prognostic marker, since it provides prognostic information over and above clinical and conventional echocardiographic risk factors. Furthermore, an impaired longitudinal deformation performance outside the culprit lesion perfusion region seems to be a paramount marker of an adverse outcome.
